# Image-Guided Superficial Radiation Therapy for Basal and Squamous Cell Carcinomas Produces Excellent Freedom from Recurrence Independent of Risk Factors

**DOI:** 10.3390/jcm13195835

**Published:** 2024-09-30

**Authors:** Rania Agha, Randy V. Heysek, David B. Vasily, Russell Rowe, Erin M. McClure, Kathryn O’Reilly, Steven Eric Finkelstein, Aaron S. Farberg

**Affiliations:** 1 Department of Dermatology, University of Illinois at Chicago, Chicago, IL 60612, USA; 2 Jesse Brown VA Medical Center, Chicago, IL 60612, USA; 3 Chicago Medical School, Rosalind Franklin University of Medicine and Science, North Chicago, IL 60064, USA; 4 Central Florida Cancer Institute, Davenport, FL 33837, USA; 5 Lehigh Valley Dermatology Associates, Ltd., Bethlehem, PA 18018, USA; david.vasily.md@lehighvalleyderm.com; 6 RealSkin Dermatology, Waco, TX 76712, USA; 7 University Hospitals Geauga Medical Center, Chardon, OH 44024, USA; 8 Cape Henlopen Dermatology, P.A., Lewes, DE 19958, USA; koreilly6@gmail.com; 9 Center of Advanced Radiation Excellence (CARE) and Radiation Oncology Research, Associated Medical Professionals of NY, Syracuse, NY 13210, USA; steven.finkelstein@ampofny.com; 10 Bare Dermatology, Dallas, TX 75235, USA; 11 University of North Texas Health Science Center, Fort Worth, TX 76107, USA

**Keywords:** non-melanoma skin cancer, image-guided superficial radiation therapy, freedom from recurrence, tumor location, head, neck, tumor stage, sex

## Abstract

**Background/Objectives**: Basal cell carcinomas (BCCs) and squamous cell carcinomas (SCCs) are non-melanoma skin cancers (NMSCs) and the most prevalent cancers in the United States. Image-guided superficial radiotherapy (IGSRT) is a relatively new treatment option that uses high-resolution dermal ultrasound integrated with superficial radiotherapy to improve tumor visualization. IGSRT is a clinically equivalent non-surgical alternative to Mohs micrographic surgery at 2 years of follow-up in early-stage NMSC, but larger cohort studies with longer follow-up periods that allow for analysis of patient outcomes by demographic and disease characteristics are needed. **Methods**: This large, retrospective cohort study was conducted to determine the effect of risk factors (tumor location, tumor stage, and sex) on 2-, 4-, and 6-year freedom from recurrence rates in 19,988 NMSC lesions treated with IGSRT, including lesions with complete treatment courses. **Results**: Overall freedom from recurrence rates were 99.68% at 2 years, 99.54% at 4 years, and 99.54% at 6 years; rates did not differ significantly by tumor location (head/neck versus other locations, *p* = 0.9) or sex (male versus female, *p* = 0.4). In contrast, there was a significant difference in freedom from recurrence rates when analyzed by tumor stage (*p* = 0.004). **Conclusions**: There was no significant effect of tumor location or sex on freedom from recurrence in IGSRT-treated NMSC. Although there was a significant difference according to tumor stage, freedom from recurrence rates exceeded 99% at all stages.

## 1. Introduction

Basal cell carcinomas (BCCs) and squamous cell carcinomas (SCCs) are non-melanoma skin cancers (NMSCs) that originate from epidermal cells and share a common epidemiology [1]. NMSCs constitute approximately one-third of all malignancies diagnosed globally, with rising incidence worldwide, primarily owing to increased exposure to ultraviolet radiation [2,3]. NMSCs are the most common cancers in the United States [4]. In 2019 in the United States, the incidences of BCC and SCC were 525 and 262 per 100,000 persons, respectively [5]; these incidences are estimated to be increasing by approximately 2% annually [4,5,6]. Most NMSCs have a good prognosis and a low risk of metastasis. However, globally, NMSCs are responsible for about 65,000 deaths per year [7]. SCC causes the majority of NMSC-related deaths. Specifically, the cancer-specific mortality ratio estimate for SCC is 2.17 versus 1.15 for BCC [8]. 

Most NMSCs are found in sun-exposed areas such as the face, head, and neck, which are considered cosmetically and functionally sensitive areas. BCC is the most common type of skin cancer (accounting for about 80% of skin cancers) and originates in the basal cells [1,9]. BCC rarely metastasizes, and mortality is, therefore, only 0.02 per 10,000 cases [7]; however, these cancers can locally invade and destroy healthy tissue, which can result in high morbidity [1]. In contrast, SCC is the second most common skin cancer and originates in the squamous cells [9]. SCC is extremely heterogeneous and categorized into many different subtypes, ranging from slow growth to aggressive, invasive tumors with high risk of spreading towards distant sites [1]. Metastatic SCC is responsible for 75% of deaths caused by NMSC, with a 10-year survival rate of 10–20% [7,10]. Squamous cell carcinoma in situ (SCCIS) is the earliest form of SCC; the cancer cells are only present in the epidermis and have not yet invaded deeper layers [9]. SCCIS can progress to invasive SCC, so early treatment is recommended [9].

The NMSC stage indicates how large the lesion is and whether there is distant metastasis; management depends on the stage at presentation [11]. The eighth edition of the American Joint Committee on Cancer (AJCC) tumor, node, and metastasis staging system has stages ranging from 1 to 4, with lower stages indicating less spread [11]. This system can be used to stage both BCC and SCC. According to the National Comprehensive Cancer Network (NCCN) and Dermatology Association of Radiation Therapy clinical guidelines, BCC and SCC can also be divided into risk groups to determine optimal treatment options [12,13,14,15]. BCCs are divided into low and high risk according to the risk of recurrence after treatment, and SCCs are divided into low, high, and very high risk according to the risk of recurrence or spread after treatment [11]. Whether a tumor is considered low-, high-, or very high-risk depends on risk factors such as tumor location, size, depth, thickness, and level of invasion; how well or ill-defined the borders of the tumor are; whether the tumor is primary versus recurrent; if the patient has a weakened immune system; if the cancer has been previously treated with radiation; the tumor growth rate and its histologic features/subtype; and whether there is perineural, lymphatic, or vascular involvement [11,12,13,14,15].

For early-stage BCC and SCC, current NCCN guidelines recommend surgical excision or Mohs micrographic surgery (MMS) [12,13]. MMS is a tissue-conserving surgical procedure commonly used to treat skin cancer when the goal is to excise high-risk BCC or SCC and/or lesions on sensitive areas like the central face, hands, feet, nipples, or genitalia [16]. Another resource for evaluating lesions for MMS is the 2012 Appropriate Use Criteria [17]. Definitive radiation therapy is recommended by the NCCN for patients who are poor surgical candidates or for those who prefer a non-surgical approach [12,13]. 

Superficial radiation therapy (SRT) is a form of radiation that uses low-energy kilovoltage photons to confine treatment to the skin [18]. Image-guided superficial radiation therapy (IGSRT), which received clearance from the United States Food and Drug Administration (FDA) in 2015, was developed to improve the precision of traditional SRT and is becoming a commonly used alternative to surgery for NMSC [19]. IGSRT uses high-resolution dermal ultrasound technology integrated with SRT to improve the visualization of skin cancers, allowing for more accurate and adaptive targeting of radiation treatment. During IGSRT, an ultrasound unit designed to detect dermatologic structures is set to a frequency of 22 MHz, which is ideal for visualizing superficial skin layers with a depth of 0–6 mm. This allows for the determination of tumor depth and the extent of the lesion beyond clinical visibility [19]. IGSRT has been shown to be a highly effective treatment option for NMSCs, at minimum, demonstrating clinical equivalence to MMS-treated early-stage NMSCs (SCCs and BCCs) [20]. Furthermore, IGSRT is an attractive treatment option for patients who prefer to avoid adverse surgical effects (e.g., hematoma, scarring, or wound dehiscence) or who have contraindications to surgery.

There is now a need for larger cohort studies with longer follow-up periods that allow for analysis of patient outcomes by specific demographic and disease characteristics. Therefore, the objective of this large, retrospective cohort study was to determine the effects of risk factors such as patient sex and tumor location and stage on freedom from recurrence in patients with IGSRT-treated NMSC.

## 2. Materials and Methods

### 2.1. IGSRT Methodology and Energy/Dose Selection Process 

The treatment methodology has been previously described in detail [19,21] and follows a general guideline (the Ladd–Yu protocol) [19] for treatment dose, energy, fractionation, and therapeutic biologic effect represented by time–dose–fractionation (TDF) calculations. A standardized protocol with a total of ~20 fractions using single energy or a sequential combination of 50 kVp, 70 kVp, or 100 kVp energy X-ray treatment is generally delivered 2–4 times per week. Treatment is preceded by daily high-resolution dermal ultrasound (HRDUS; manufactured by Cortex Technology Derma-Scan C Ultrasound System in Hadsund, Denmark) to assess/confirm tumor configuration/location and detect changes, which may indicate a prescription change as necessary, with adaptive radiation treatment planning. HRDUS was also performed during initial simulation for treatment planning purposes, as well as at follow-up evaluations after treatment course completion to evaluate response. 

### 2.2. Tumor Configuration and Depth Determination 

HRDUS uses a non-invasive 20–22 MHz ultrasound with a Doppler component probe that is intrinsic to the IGSRT unit (Sensus SRT-100 Vision). This allows for visualization 0–10 mm into the skin structure, including visualization of the epithelium and papillary and, sometimes, down to the reticular dermis, depending on the anatomic location and skin thickness. Such high-resolution/high-frequency ultrasound allows for clear visualization of normal skin anatomy and the disrupting tumor, which is hypoechoic without Doppler color speckles and allows for precise visualization, measurement, and capture of the exact depth of penetration, permitting the clinician to perform adaptive radiation therapy planning during a course of care. The width and configuration of the tumor can also be easily discerned with HRDUS and are integral to localization and treatment planning, reducing the risk of anatomical miss and misadministration. Radiation therapist technologists are trained to hold the ultrasound probe in a consistent direction, scanning the tumors and the field margin in the same orientation every time. To assessing lesion depth, lesions are measured from the skin surface to the deepest portion of the tumor with a straight line. These techniques improve the reliability and reproducibility of tumor measurements and treatment administration.

### 2.3. Data Collection 

Data collection followed a process described in previously published studies [19,21]. Records of all NMSC lesions treated with IGSRT at multiple institutions across the continental United States between 2016 and 2023, regardless of whether treatment was completed, were retrospectively gathered. In total, 19,988 lesions were included in this cohort. Only NMSCs of the BCC, SCC, and SCCIS types were included. Exclusion criteria included cases missing pertinent documentation (i.e., treatment chart, simulation statistics like time, dose, and fractionation); stage 3 tumors with deep invasion, cortical erosion, or perineural invasion; and stage 4 tumors. Patient characteristics and treatment parameters for NMSC lesions were extracted manually and accessed electronically from written and electronic medical records (EMRs) for all institutions. Additional data from the EMR, including race, ethnicity, past medical history, medications, follow-up dates, and mortality status/expiratory dates, were “data-scraped” with algorithmic programming conducted by Sympto Health, Inc., San Francisco, CA, USA.

### 2.4. Statistical Analysis 

Detailed logs of NMSC recurrences were maintained by the dermatology practices’ radiation therapists. These logs were used to quantify recurrence events. Recurrences can be reported at any follow-up appointment. The typical follow-up schedule consists of 2- and 6-week post IGSRT visits where ultrasound is performed, a dermatologist evaluates the treatment site clinically, and photos are taken. Patients are then seen for a 3-month skin check, followed by routine follow-up appointments. If an NMSC is biopsied within the treatment area and is the same histologic type as the originally treated cancer, it is logged as a recurrence.

Freedom from recurrence was estimated using the Kaplan–Meier method. Groups were compared with respect to freedom from recurrence using the log rank test. 

### 2.5. Ethics 

The ethics committee/Institutional Review Board (IRB) of WIRB-Copernicus Group. Inc. (WCG, Princeton, NJ, USA) waived ethical approval for this work. The dataset was de-identified prior to analysis and all data personnel adhered to the Health Insurance Portability and Accountability Act (HIPAA) and ethical standards to protect patient information. This study was granted exemption status on 18 April 2024.

## 3. Results

### 3.1. Patient and Disease Characteristics

A total of 19,998 lesions were evaluated using an intention-to-treat analysis. Demographic and disease characteristics of patients are summarized in Table 1. As shown, patients were mostly male (61.7%) and aged ≥65 years (84.2%). Most lesions were located in the head or neck (63.7%), which are known high-risk BCC and SCC locations [1]. Of these, the majority were on the nose (17.3%) and cheek (14.8%). Most lesions were categorized as early-stage per the AJCC 8th edition staging system (stage 0 was added to this analysis to represent SCCIS), i.e., stage 0 (23.4%) or 1 (65.7%). With regard to histology, lesions were diagnosed as BCC in 49.5% of cases, SCC in 26.4%, SCCIS in 23.2%, and as a combination of two or more NMSC types in 1.0%.

### 3.2. Freedom from Recurrence Rates by Tumor Location

**Figure 1:**
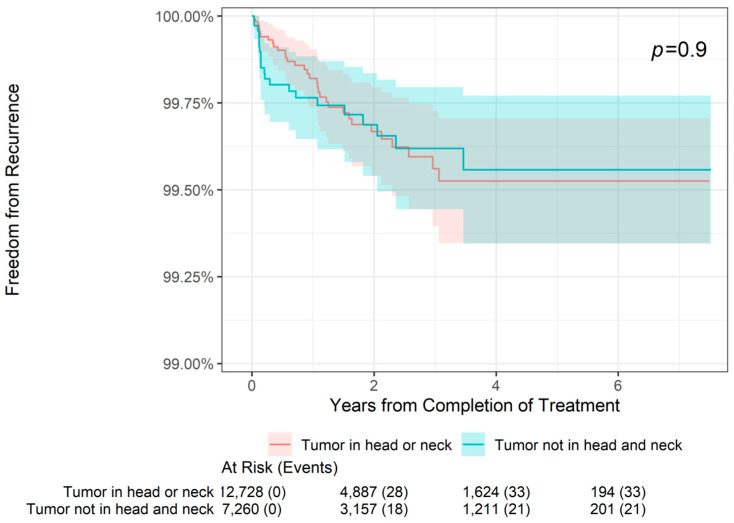
Freedom from recurrence over time of non-melanoma skin cancer treated with image-guided superficial radiation therapy by tumor location.

The 2-, 4-, and 6-year freedom from recurrence rates are presented by tumor location in Table 2. As shown, the overall 2-year freedom from recurrence rate in the total sample was 99.68%, with freedom from recurrence in 99.67% of patients with both tumors on the head or neck and those with tumors in other locations (i.e., locations other than the head or neck). Similarly, the overall 4-year freedom from recurrence rate was 99.54%, with freedom from recurrence in 99.53% of patients with head or neck tumors and 99.56% of patients with tumors in other locations. Finally, the overall 6-year freedom from recurrence rate was 99.54%, with freedom from recurrence in 99.53% of patients with head or neck tumors and 99.56% of patients with tumors in other locations. As shown in Figure 1, there was no statistically significant difference in freedom from recurrence when stratifying patients by tumor location (tumor on head or neck versus tumor not on head or neck, *p* = 0.9). For patients with head or neck tumors and for those with tumors in other locations, freedom from recurrence decreased slightly from 2- to 4-year follow-up but remained stable at 6 years.

### 3.3. Freedom from Recurrence Rates by Tumor Stage

**Figure 2:**
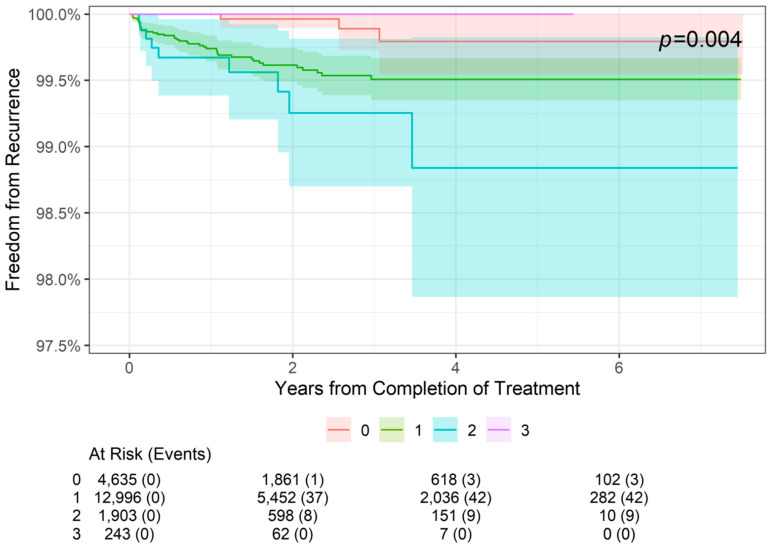
Freedom from recurrence over time of non-melanoma skin cancer treated with image-guided superficial radiation therapy by tumor stage (AJCC 8th edition staging).

The 2-, 4-, and 6-year freedom from recurrence rates are presented by tumor stage in Table 3. As shown, 2-year freedom from recurrence rates were more than 99% for lesions of all stages (99.96% for stage 0 lesions (SCCIS), 99.62% for stage 1 lesions, and 99.25% for stage 2 lesions). Similarly, 4-year and 6-year freedom from recurrence rates were 99.79% for stage 0 lesions, 99.51% for stage 1 lesions, and 98.94% for stage 2 lesions. The difference among the four stages was statistically significant (*p* = 0.004; Figure 2). For patients with stage 0 to stage 2 NMSC, freedom from recurrence rates worsened from 2 to 4 years but remained stable at 6 years. Stage 3 data are from lesions 4 cm in diameter or larger in, and no recurrences in this group were observed at 2- or 4-year follow-up; however, data were not available at the 6-year point.

### 3.4. Freedom from Recurrence Rates by Patient Sex

**Figure 3:**
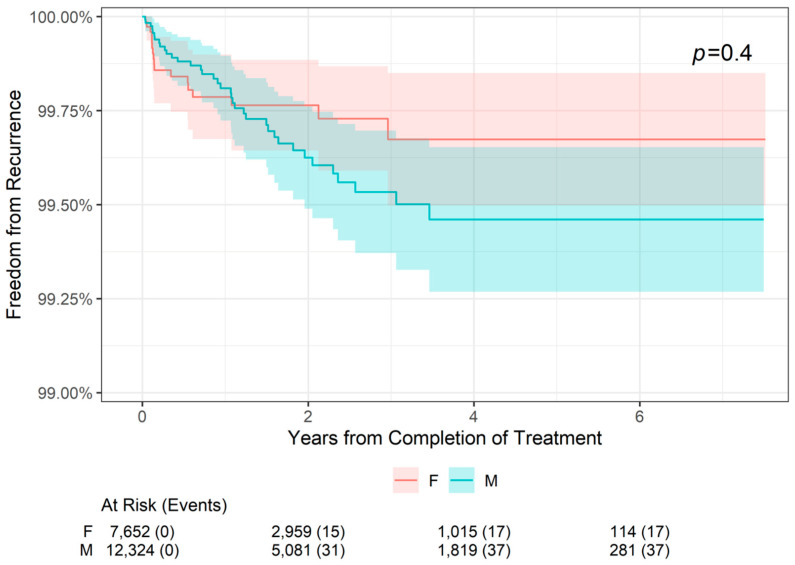
Freedom from Recurrence over time of non-melanoma skin cancer treated with image-guided superficial radiation therapy by patient sex.

The 2-, 4-, and 6-year freedom from recurrence rates are presented by patient sex in Table 4. Freedom from recurrence rates were slightly higher for female patients compared with male patients at 2 years (99.76% versus 99.63%, respectively) and at 4 and 6 years (99.67% versus 99.46%, respectively). However, as shown in Figure 3, there was no statistically significant difference in freedom from recurrence when stratifying patients by sex (*p* = 0.4). For both male and female patients, freedom from recurrence decreased slightly from 2- to 4-year follow-up and remained stable at 6 years.

## 4. Discussion

The major finding from this large (19,988 lesions), retrospective cohort study is that the effects of IGSRT on NMSC are not significantly impacted by known risk factors such as tumor location or patient sex. For all patients, overall freedom from recurrence rates were 99.68% at 2 years, 99.54% at 4 years, and 99.54% at 6 years, and there was no statistically significant difference in freedom from recurrence when stratifying patients by tumor location (head or neck tumors versus tumors in other locations, *p* = 0.9) or patient sex (*p* = 0.9). Head/neck NMSC tumor sites are considered to be relatively high-risk for recurrence, so it is important that IGSRT has been shown to be equivalently effective for these tumors compared to non-head/neck tumors. Future analysis of other high-risk NMSC tumor sites (hands, feet, pretibial, and anogenital area) would help further guide optimal treatment decisions with respect to the anatomical location of the disease. Male sex has been associated with worse NMSC outcomes [1]. However, with respect to recurrence rates after MMS, a paper on 163 patients with NMSCs found no association between patient sex and 5-year recurrence (which was 1.84% overall) [22]. Similarly, a paper on 154 patients that had MMS for their BCCs had a 4-year overall recurrence rate of 1.9%, which was not impacted by patient sex (*p* = 0.7) [23]. A study on 237 MMS-treated SCCs found no statistically significant effect of sex or tumor location on recurrence [24]. These data suggest that the decision to pursue IGSRT or MMS should not be dependent on patient sex. 

In contrast, the effects of IGSRT on NMSC were affected by tumor stage. Although overall freedom from recurrence was more than 99% for all stages (AJCC 8th edition stages 0–3), the difference among the four stages was statistically significant (*p* = 0.004). The maintenance of more than 99% freedom from recurrence across all evaluated tumor stages supports this modality as a highly effective treatment for these cancers. As expected, freedom from recurrence decreases as tumor stage increases. Interestingly, stage 3 tumors had 100% freedom from recurrence at 2- and 4-year follow-up, which is better than the lower-stage tumors in this cohort. However, this is likely due to the relatively small sample size of stage 3 tumors (*n* = 249) rather than IGSRT being more effective in these higher-risk tumors. Notably, this study did not evaluate the outcomes of high-risk stage 3 tumors or stage 4 tumors, as these are outside the scope of definitive treatment by IGSRT. Unfortunately, the literature on the recurrence rates of MMS-treated NMSCs stratified by tumor stage is lacking, so comparisons cannot be made.

In this cohort of 19,988 lesions, 54 recurred. Supplemental Table S1 details the characteristics of the recurrent lesions. The most common recurrent site was the ear, which is known to be more prone to recurrence following surgical intervention [25]. Interestingly, BCCs comprised nearly 60% of the recurrent tumors. While BCCs are less aggressive than SCCs, it is possible that subclinical spread of BCCs contributed to this finding [26].

These findings demonstrate that IGSRT is a viable therapeutic option for patients with both high- and low-risk NMSC and support previous research that found that IGSRT is a safe, well-tolerated therapy that demonstrates excellent local tumor control, absolute lesion control [19], and superior 2-year recurrence probabilities compared to MMS [20]. 

As a treatment, IGSRT has several advantages. For example, it is a remarkably well-tolerated treatment, with the majority of adverse events reported as low-grade cutaneous toxicities (i.e., erythema, xerosis, and dry desquamation), which typically resolve within several weeks after treatment completion [20]. The procedure is also relatively quick to administer; visits are 10–15 min long, each lesion treatment is less than 1 min, and up to 4 lesions can be treated in a single visit, whereas a typical MMS takes 2–4 h [20]. Furthermore, IGSRT is suitable for patients with contraindications for surgery (such as allergies to local anesthetics, severe cardiac disease, or coagulopathy) or those who prefer not to undergo surgical procedures and provides functional preservation and favorable cosmetic outcomes (i.e., there is a low risk of scarring from this non-invasive procedure) [20]. This is particularly important, as NMSCs most commonly occur in cosmetically and functionally sensitive areas such as the face, head, and neck.

Various studies have evaluated the outcomes of NMSCs treated by IGSRT. For example, in one study, IGSRT demonstrated 99.3% local tumor control in 2,917 lesions after an average of 7.1 weeks of treatment and an average of 69.8 months of follow-up [19]. In another study, IGSRT demonstrated 99.7% absolute lesion control and 99.6% stable control in 1,899 lesions after an average of 7.5 weeks of treatment with over 12 months of follow-up [21]. A couple of meta-analyses also support IGSRT as an effective treatment for early-stage NMSC. They found that IGSRT is superior to traditional non-image-guided SRT and non-image-guided radiotherapy overall and in all cancer subtypes when stratified by histology [27,28].

One important limitation of this study is its retrospective design, as randomized controlled trials (RCTs) are the gold standard for treatment comparisons and can generate cause–effect data, whereas retrospective studies only establish associations. Specific limitations of retrospective study designs include missing data, as data are retrieved from a clinical database rather than a database that was pre-emptively designed with the specific study in mind. Additionally, non-responders or patients experiencing adverse effects are more likely to be lost to follow-up, which contributes to bias [29]. Another limitation is that the documentation of recurrences by practices depends on dermatologists documenting and reporting recurrences in a tumor recurrence registry. It is possible that some patients are referred immediately by their dermatologist to Mohs or surgical oncology for the treatment of their recurrence and that the tumor recurrence registry is not notified and, thus, the recurrence is not documented. However, the participating dermatology practices were requested to review the tumor recurrence registry prior to data extraction to verify that all pertinent information was captured, including recurrences requiring further interventions. 

Future RCTs would be helpful to improve the quality of the evidence behind IGSRT. In addition, further retrospective analyses would be useful to investigate the impact of other demographic and disease characteristics (e.g., age, tumor histology, tumor depth, and comorbidities) to gain further insight into the patient populations that might gain the most benefit from IGSRT. Lastly, an analysis utilizing the genomic-adjusted radiation dose (GARD) model to personalize radiation doses to individual patients with NMSCs may further optimize the outcomes and side effects associated with IGSRT [30].

## 5. Conclusions

In summary, this is the first large, retrospective cohort study of patients with NMSCs treated with IGSRT to evaluate and compare freedom from recurrence by tumor risk factors. Overall, this study found that freedom from recurrence rates do not vary significantly among patients with high-risk tumors of the head or neck versus other lower-risk locations or between male and female patients. Although there were statistically significant differences by tumor stage, overall freedom from recurrence rates were above 99% for patients with tumors of all stages. These results suggest that IGSRT is a viable first-line therapeutic option for patients diagnosed with early-stage high- and low-risk NMSC.

## Figures and Tables

**Table 1:** Patient and disease characteristics.

Characteristic	All Lesions (*n* = 19,998)	BCC (*n* = 9885)	SCC (*n* = 5270)	SCCIS (*n* = 4635)	Two or More NMSC Types (*n* = 198)
Age					
Median (IQR)	74.9 (68.2, 81.7)	73.3 (66.2, 80.6)	76.7 (70.2, 82.9)	75.7 (69.7, 82.1)	78.6 (72.2, 84.8)
<65 years	3152 (15.8)	2042 (20.7)	547 (10.4)	545 (118)	18 (9.1)
≥65 years	16,836 (84.2)	7843 (79.3)	4723 (89.6)	4090 (88.2)	180 (90.9)
Sex					
Female	7652 (38.3)	3895 (39.4)	1925 (36.6)	1766 (38.1)	66 (33.3)
Male	12,324 (61.7)	5985 (60.6)	3341 (63.4)	2866 (61.9)	132 (66.7)
Unknown	12	5	4	3	0
Tumor location					
Head/neck	12,728 (63.7)	7098 (71.8)	2784 (52.8)	2693 (58.1)	153 (77.3)
Ear	1586 (7.9)	812 (8.2)	433 (8.2)	322 (6.9)	19 (9.6)
Scalp	1205 (6.0)	251 (2.5)	418 (7.9)	510 (11.0)	26 (13.1)
Forehead	1672 (8.4)	863 (8.7)	361 (6.9)	428 (9.2)	20 (10.1)
Temple	559 (2.8)	285 (2.9)	134 (2.5)	131 (2.8)	9 (4.5)
Orbit/eyelid	105 (0.5)	81 (0.8)	10 (0.2)	14 (0.3)	0 (0.0)
Nose	3282 (16.4)	2537 (25.7)	372 (7.1)	338 (7.3)	35 (17.7)
Cheek	784 (13.9)	1355 (13.7)	730 (13.9)	668 (14.4)	31 (15.7)
Mucosal lip	47 (0.2)	14 (0.1)	24 (0.5)	9 (0.2)	0 (0.0)
Chin/mandible	117 (0.6)	91 (0.9)	18 (0.3)	8 (0.2)	0 (0.0)
Neck	674 (3.4)	403 (4.1)	124 (2.4)	139 (3.0)	8 (4.0)
Other	97 (3.5)	406 (4.1)	160 (3.0)	126 (2.7)	5 (2.5)
Extremities	4125 (20.6)	1080 (10.9)	1791 (34.0)	1228 (26.5)	26 (13.1)
Shoulder	17 (0.1)	10 (0.1)	4 (0.1)	3 (0.1)	0 (0.0)
Hand/foot	522 (2.6)	38 (0.4)	270 (5.1)	210 (4.5)	4 (2.0)
Other	3586 (17.9)	1032 (10.4)	1517 (28.8)	1015 (21.9)	22 (11.1)
Trunk	817 (4.1)	528 (5.3)	126 (2.4)	157 (3.4)	6 (3.0)
Chest	279 (1.4)	138 (1.4)	66 (1.3)	73 (1.6)	2 (1.0)
Back	393 (2.0)	292 (3.0)	43 (0.8)	56 (1.2)	2 (1.0)
Other	145 (0.7)	98 (1.0)	17 (0.3)	28 (0.6)	2 (1.0)
Stage ^a^					
0	4635 (23.4)	0 (0.0)	0 (0.0)	4635 (100.0)	0 (0.0)
1	12,996 (65.7)	8436 (86.4)	4410 (85.0)	0 (0.0)	150 (76.9)
2	1903 (9.6)	1176 (12.1)	698 (13.5)	0 (0.0)	23 (14.9)
3	249 (1.2)	147 (1.5)	80 (1.5)	0 (0.0)	16 (8.2)
Unknown	211	126	82	0	3

All data presented as *n* (%) unless otherwise indicated. ^a^ AJCC 8th edition staging used. Abbreviations: AJCC, American Joint Committee on Cancer; BCC, basal cell carcinoma; IQR, interquartile range; NMSC, non-melanoma skin cancer; SCC, squamous cell carcinoma; SCCIS, squamous cell carcinoma in situ.

**Table 2:** Freedom from recurrence rates by tumor location.

Group	2-Year Freedom from Recurrence	4-Year Freedom from Recurrence	6-Year Freedom from Recurrence
Overall	8044 (99.68)	2835 (99.54)	395 (99.54)
Tumor on head or neck	4887 (99.67)	1624 (99.53)	194 (99.53)
Tumor not on head or neck	3157 (99.69)	1211 (99.56)	201 (99.56)

All data presented as *n* (%).

**Table 3:** Freedom from recurrence rates by tumor stage (intention-to-treat cohort).

Stage ^a^	2-Year Freedom from Recurrence	4-Year Freedom from Recurrence	6-Year Freedom from Recurrence
Overall	8044 (99.68)	2835 (99.54)	395 (99.54)
Stage 0	1861 (99.96)	618 (99.79)	102 (99.79)
Stage 1	5452 (99.62)	2036 (99.51)	282 (99.51)
Stage 2	598 (99.25)	151 (98.84)	10 (98.84)
Stage 3	63 (100)	7 (100)	N/A

All data presented as *n* (%). ^a^ AJCC 8th edition staging used. Abbreviations: AJCC, American Joint Committee on Cancer; N/A, not applicable.

**Table 4:** Freedom from recurrence rates by patient sex.

Group	2-Year Freedom from Recurrence	4-Year Freedom from Recurrence	6-Year Freedom from Recurrence
Overall	8044 (99.68)	2835 (99.54)	395 (99.54)
Female	2959 (99.76)	1015 (99.67)	114 (99.67)
Male	5081 (99.63)	1819 (99.46)	281 (99.46)

All data presented as *n* (%).

## Data Availability

The data presented in this study are available upon request from the corresponding author.

## Supplementary Materials

The following supporting information can be downloaded at: https://www.mdpi.com/article/10.3390/jcm13195835/s1, Table S1: Recurrent Tumor Characteristics.
