# Supplementary material for: Image-Guided Superficial Radiation Therapy for Basal and Squamous Cell Carcinomas Produces Excellent Freedom from Recurrence Independent of Risk Factors
Source: J Clin Med. 2024 Sep 30;13(19):5835. doi: 10.3390/jcm13195835 (PMC11477103; doi:10.3390/jcm13195835)
Supplement: Supplementary file 1 [file jcm-13-05835-s001.zip › jcm-3214397-supplementary.pdf]

Supplemental Table S1. Recurrent Tumor Characteristics

Characteristic	All Lesions (n=19998)	Non-recurrent lesions (n= 19934)	Recurred lesions (n=54)
Age			
Median (IQR)	74.9 (68.2, 81.7)	74.9 (68.2, 81.6)	74.4 (68.4, 81.2)
<65 years	3152 (15.8)	3145 (15.8)	7 (13.0)
≥65 years	16836 (84.2)	16789 (84.2)	47 (87.0)
Sex			
Female	7652 (38.3)	7635 (38.3)	17 (31.5)
Male	12324 (61.7)	12287 (61.7)	37 (68.5)
Unknown	12	12	0
Histology			
BCC	9885 (49.5)	9853 (49.4)	32 (59.3)
SCC	5270 (26.4)	5251 (26.3)	19 (35.2)
SCCIS	4635 (23.2)	4632 (23.2)	3 (5.6)
2 or more NMSCs	198 (1.)	198 (1.0)	0 (0.0)
Tumor location			
Head/neck	12728 (63.7)	12695 (63.7)	33 (61.1)
Ear	1,586 (7.9)	1577 (7.9)	9 (16.7)
Scalp	1205 (6.0)	1201 (6.0)	4 (7.4)
Forehead	1672 (8.4)	1669 (8.4)	3 (5.6)
Temple	559 (2.8)	557 (2.8)	2 (3.7)
Orbit/Eyelid	105 (0.5)	104 (0.5)	1 (1.9)
Nose	3282 (16.4)	3274 (16.4)	8 (14.8)
Cheek	2784 (13.9)	2782 (14.0)	2 (3.7)
Mucosal lip	47 (0.2)	47 (0.2)	0 (0.0)
Chin/Mandible	117 (0.6)	116 (0.6)	1 (1.9)
Neck	674 (3.4)	673 (3.4)	1 (1.9)
Other	697 (3.5)	695 (3.5)	2 (3.7)
Extremities	4125 (20.6)	4115 (20.6)	10 (18.5)
Hand/Foot	522 (2.6)	520 (2.6)	2 (3.7)
Shoulder	17 (0.1)	17 (0.1)	0 (0.0)
Other	3586 (17.9)	3578 (17.9)	8 (14.8)
Trunk	817 (4.1)	816 (4.1)	1 (1.9)
Chest	279 (1.4)	278 (1.4)	1 (1.9)
Back	393 (2.0)	393 (2.0)	0 (0.0)
Other	145 (0.7)	145 (0.7)	0 (0.0)
Stage[a]			
0	4635 (23.4)	4632 (23.5)	3 (5.6)
1	12996 (65.7)	12954 (65.7)	42 (77.8)
2	1903 (9.6)	1894 (9.6)	9 (16.7)

3	249 (1.2)	243 (1.2)	0 (0.0)
Unknown	211	211	0

All data presented as n (%) unless otherwise indicated. [a]AJCC 8th edition staging used. Abbreviations: AJCC, American Joint Committee on Cancer; BCC, basal cell carcinoma; IQR, interquartile range; NMSC, non-melanoma skin cancer; SCC, squamous cell carcinoma; SCCIS, squamous cell carcinoma in-situ.
